# Underuse of osteoporosis treatments before and after hip fracture: longitudinal findings from the Gruppo Italiano di Ortogeriatria (GIOG 2.0) study

**DOI:** 10.3389/fragi.2026.1837212

**Published:** 2026-05-29

**Authors:** Maria Cristina Ferrara, Wenxiang Guo, Eleonora Cucini, Alice Margherita Ornago, Elena Tassistro, Elena Pinardi, Alberto Finazzi, Paolo Mazzola, Chukwuma Okoye, Maria Grazia Valsecchi, Chiara Mussi, Giuseppe Sergi, Andrea Ungar, Raffaele Antonelli Incalzi, Stefano Volpato, Giuseppe Bellelli

**Affiliations:** 1 School of Medicine and Surgery, University of Milano-Bicocca, Monza, Italy; 2 Department of Neurobiology, Care Sciences and Society, Aging Research Center, Karolinska Institutet and Stockholm University, Stockholm, Sweden; 3 Biostatistics and Clinical Epidemiology, Fondazione IRCCS San Gerardo dei Tintori, Monza, Italy; 4 Bicocca Center of Bioinformatics, Biostatistics and Bioimaging (B4 Centre), School of Medicine and Surgery, University of Milano-Bicocca, Monza, Italy; 5 Acute Geriatric Unit, Fondazione IRCCS San Gerardo dei Tintori, Monza, Italy; 6 Department of Biomedical, Metabolic and Neural Sciences, University of Modena and Reggio Emilia, Modena, Italy; 7 Department of Medicine, Geriatric Unit, University of Padua, Padua, Italy; 8 Department of Geriatrics, Careggi University Hospital, University of Florence, Florence, Italy; 9 Fondazione Policlinico Universitario Campus Bio-Medico, Roma, Italy; 10 Research Unit of Internal Medicine, Department of Medicine and Surgery, Università Campus Bio-Medico di Roma, Roma, Italy; 11 Department of Medical Science, University of Ferrara, Ferrara, Italy

**Keywords:** hip fracture, older adults, orthogeriatric, osteoporosis treatment, treatment gap

## Abstract

**Background:**

Osteoporosis is a major cause of fragility fractures, with hip fracture (HF) representing the most severe complication in older adults. However, national data on osteoporosis treatment use before and after hip fracture in Italy are limited.

**Methods:**

This multicenter prospective study included individuals aged ≥65 years admitted for HF between July 2019 and June 2024 at 12 orthogeriatric centers. Osteoporosis treatments (vitamin D, calcium, antiresorptive, anabolic, dual effect agents) were recorded at admission, discharge, and 120- and 365-day follow-ups. Patients were classified as “Never users” if untreated throughout the study period or “Ever users” if treated at least once. Multivariate logistic regression identified factors independently associated with lack of treatment.

**Results:**

Among 1,012 patients (median age 83 years; 77.2% female), 85.6% were untreated at admission; this proportion dropped to 34.4% at discharge but rose again to 50% at 1 year. Vitamin D and calcium were the predominant treatments, while antiresorptive, anabolic, and dual effect agents were underused. Overall, 22% of patients were classified as “Never users”. They were generally older, more often male, and had higher comorbidity, dementia, frailty, and mobility limitations. Only male sex was independently associated with lack of treatment (Odds Ratio 2.37, 95% Confidence Intervals 1.59–3.55).

**Conclusion:**

Despite clinical guideline recommendations, osteoporosis treatment remains suboptimal among older HF patients in Italy, especially in male patients. Findings support the need for national programs and multidisciplinary strategies to reduce the treatment gap and improve long-term management.

## 1 Introduction

Osteoporosis is a major global health challenge due to its strong association with fragility fractures, a condition whose burden exceeds that of chronic obstructive pulmonary disease and ischemic stroke (LeBoff et al., 2022; Willers et al., 2022). In Italy, an estimated 560,000 fragility fractures occurred in 2017, generating €9.4 billion in healthcare costs, a figure projected to reach €11.9 billion by 2030 (Willers et al., 2022). Among these, hip fractures (HF) are the most serious, carrying severe consequences for both patients and healthcare systems, including increased mortality, greater rehabilitation needs, loss of independence in activities of daily living, and often a persistent fear of falling (Guzon-Illescas et al., 2019; Dyer et al., 2016; Veronese and Maggi, 2018; Gadhvi et al., 2023).

Pharmacological management of osteoporosis includes antiresorptive drugs (e.g., bisphosphonates, denosumab), anabolic agents (e.g., teriparatide), or dual effect agents (e.g., romosozumab), alongside vitamin D and/or calcium supplementation (Pavone et al., 2017; Morin et al., 2023; Kanis et al., 2020). These treatments have demonstrated cost-effectiveness in multiple randomized controlled trials and real-world studies (O’Kelly et al., 2022; Saag et al., 2017), and are recommended by international guidelines and expert consensus statements (Pavone et al., 2017; Morin et al., 2023; Fuggle et al., 2024; Kanis et al., 2019). Nonetheless, a 2020 study conducted across 153 General Practitioners (GPs) sites in eight countries (e.g., Belgium, France, Germany, Ireland, Poland, Slovakia, Switzerland, and the United Kingdom) found that approximately 75% of those eligible for treatment in primary care were not receiving osteoporosis treatment (McCloskey et al., 2021). A similar gap is observed in Italy, where the 2023 OSMED report indicated that only about one in ten adults (median age 69 years) had received at least one treatment (AIFA, 2023). However, no national data are available on the magnitude of this gap among HF patients. This is particularly concerning, given the projected growth of the aging population in our country and the expected rise in both osteoporosis prevalence and HF incidence (Willers et al., 2022; Ström et al., 2011).

This study aimed to assess the use of osteoporosis treatments before hospitalization, at discharge, and over 1 year in older adults undergoing HF surgery at centers within the Gruppo Italiano di OrtoGeriatria (GIOG) network. As a secondary aim, we investigated the clinical and socio-demographic factors associated with lack of treatment across all time points.

## 2 Methods

### 2.1 Study population

The data were prospectively collected between July 2019 and June 2024 within the GIOG 2.0 study (Gandossi et al., 2023). GIOG 2.0 is a multicenter, prospective study enrolling individuals aged 65 and older who were consecutively admitted for HF to 12 orthogeriatric centers participating in the Italian network “Gruppo Italiano di OrtoGeriatria” (GIOG). GIOG 2.0 was approved by the Ethics Committee Brianza Institutional Review Board on 12 April 2019, and all participants provided written informed consent.

Patients alive and with available data on osteoporosis therapy at four time points (i.e., hospital admission, discharge, follow-up at 120 days, and at 365 days from surgery) were included in this study. Subsequently, thirteen patients were excluded because more than 25% of the variables required for the 24-item pre-fracture Frailty Index used in the present study were missing.

### 2.2 Osteoporosis treatments

We analyzed the use of supplements (vitamin D and calcium), and active therapies, including antiresorptive, anabolic, and dual effect agents at four specific time points. Specifically, their use was evaluated at admission (self-reported pre-fracture treatment), at discharge (treatment prescribed during hospital stay collected from discharge medical record), and at the 120- and 365-day follow-up (self-reported treatment). In case of dementia or disability, the information at admission and follow-ups were collected through proxy interviews. Patients who did not receive any osteoporosis treatment across all time points were classified as “Never users”, whereas “Ever users” were defined as those who received at least one treatment at one or more time points.

### 2.3 Sociodemographic and medical data

The data collected within the GIOG 2.0 study include anamnestic and clinical information spanning the entire hospitalization period. Sociodemographic and functional data included sex, age, living situation at the time of admission, pre-fracture ambulation level (assessed using the Standardized Audit of Hip Fracture In Europe [SAHFE] tool (Parker et al., 1998)), pre-fracture functional status (assessed using the Katz index for Activities of Daily Living, ADLs (Katz et al., 1963)), and a pre-fracture 24-item Frailty Index (FI) built according to Theou et al. indications (Supplementary Table S1) (Theou et al., 2023). Clinical variables were also recorded, including comorbidity (modified Charlson’s Comorbidity Index, mCCI (Charlson et al., 1987), calculated without age and dementia), pre-fracture diagnosis of dementia, delirium (assessed using the 4AT scale during both the pre- and post-operative phases (Bellelli et al., 2014)), as well as the type of HF (intracapsular vs. extracapsular), type of surgery (endoprosthesis; intramedullary nail or internal fixation with screws; total hip replacement), and the time-to-surgery (classified as ≤48 h vs. > 48 h according to the literature (Seong et al., 2020; Klestil et al., 2018)). Lastly, the length of stay and the destination at discharge were also collected.

### 2.4 Statistical analysis

Baseline sample characteristics are presented as medians with the first and third quartiles (Q1-Q3) for continuous, non-normally distributed variables and as counts and percentages for categorical variables. For comparisons across groups, the Kruskal-Wallis test was used for continuous variables, while the Chi-square test was used for categorical variables. The association between socio-demographic and clinical factors and the absence of osteoporosis treatment (i.e., Never users) was examined using multivariable logistic regression models. Variables significant in univariate analyses were included as covariates, except for those already accounted for in the Frailty Index. Multicollinearity was assessed (using variance inflation factors), and no significant collinearity was observed. The strength of these associations was expressed through Odds Ratios (ORs) and 95% Confidence Intervals (95% CIs). Statistical significance was set at p-value < 0.05. Statistical analyses were performed using R software version 4.4.1 (https://www.r-project.org/).

## 3 Results

Table 1 shows the main characteristics of the study populations, overall and by osteoporosis treatment use. The study included 1,012 patients with a median age of 83 years (Q1-Q3: 78–88), predominantly females (77.2%) and primarily residing at home (96.6%). The median mCCI was 1 (Q1-Q3: 0–3), and 237 (23.5%) patients had a diagnosis of dementia. Prior to fracture, functional status was relatively preserved, with an overall median ADL score of 6 (Q1-Q3: 4–6) and frailty was mild, as shown by a median FI of 0.12 (Q1-Q3: 0.05–0.25). Furthermore, 48.1% of patients were able to ambulate independently, 203 (20.1%) required aids for outdoor ambulation, 301 (29.9%) were limited to indoor ambulation, and 27 (2.3%) were non-ambulatory. Extracapsular HFs accounted for 60.9% of cases, and 77% of surgeries were performed within 48 h. Intramedullary nailing was the most common surgical procedure, performed in 58% of patients, followed by endoprosthesis in 25.1%. The prevalence of delirium was 12.4%.

**TABLE 1:** Characteristics of the study population: overall and by osteoporosis treatment use (Ever vs. Never users).

Variables	Overall (N = 1,012)	Ever users (N = 789, 78.0%)	Never users (N = 223, 22.0%)	p-value
Females, n (%)	781 (77.2)	635 (80.5)	146 (65.5)	<0.001
Age (years), median (Q1-Q3)	83.0 (78.0–88.0)	83.0 (77.0–88.0)	85.0 (79.0–89.0)	0.004
Domiciliation at admission, n (%)	​	​	​	0.115
*Home*	253 (25.0)	201 (25.5)	52 (23.3)	​
*Home with family member/caregiver*	725 (71.6)	557 (70.6)	168 (75.3)	​
*Nursing home*	34 (3.4)	31 (3.9)	3 (1.3)	​
Modified charlson comorbidity index^a^, median (Q1-Q3) (N = 945)	1.0 (0.0–3.0)	1.0 (0.0–2.0)	1.0 (0.0–3.0)	0.006
Dementia, n (%) (N = 1,009)	237 (23.5)	166 (21.1)	71 (31.8)	0.001
Activities of daily living score, median (Q1-Q3) (N = 981)	6.0 (4.0–6.0)	6.0 (4.0–6.0)	6.0 (4.0–6.0)	0.879
Frailty index, median (Q1-Q3)	0.12 (0.05–0.25)	0.11 (0.05–0.24)	0.14 (0.06–0.27)	0.015
SAHFE^b^, n (%) (N = 1,008)	​	​	​	0.016
*Able to walk independently*	485 (48.1)	392 (49.9)	93 (41.9)	​
*Able to walk outdoor (with aids)*	203 (20.1)	155 (19.7)	48 (21.6)	​
*Able to walk only indoor*	301 (29.9)	229 (29.1)	72 (32.4)	​
*Not able to walk*	19 (1.9)	10 (1.3)	9 (4.1)	​
Type of fracture, n (%)	​	​	​	0.014
*Intracapsular-subcapital*	396 (39.1)	325 (41.2)	71 (31.8)	​
*Extracapsular*	616 (60.9)	464 (58.8)	152 (68.2)	​
Type of surgery, n (%)	​	​	​	0.564
*Endoprothesis*	254 (25.1)	204 (25.9)	50 (22.4)	​
*Intramedullary nail/internal fixation with screws*	587 (58.0)	450 (57.0)	137 (61.4)	​
*Total hip replacement*	162 (16.0)	127 (16.1)	35 (15.7)	​
*Other*	9 (0.9)	8 (1.0)	1 (0.4)	​
Surgery within 48 h, n (%) (N = 977)	752 (77.0)	593 (77.7)	159 (74.3)	0.338
Delirium pre- and post- surgery, n (%) (N = 917)	114 (12.4)	88 (12.3)	26 (12.8)	0.949
Destination at discharge, n (%) (N = 1,002)	​	​	​	<0.001
*Home*	388 (38.7)	273 (34.8)	115 (52.8)	​
*Rehabilitation*	529 (52.8)	446 (56.9)	83 (38.1)	​
*Nursing home*	62 (6.2)	45 (5.7)	17 (7.8)	​
*Other wards*	23 (2.3)	20 (2.6)	3 (1.4)	​

^a^ Calculated excluding age and dementia.

^b^ Standardized Audit of Hip Fracture in Europe.

At discharge, 38.7% of patients returned home, while 52.8% were transferred to rehabilitation centers and 6.2% to nursing homes. The remaining 2.3% were transferred to other hospital wards.

Overall, 223 (22%) patients were classified as “Never users”. In comparison to “Ever users”, they were older (median 85 vs. 83 years, p = 0.004), less frequently female (65.5% vs. 80.5%, p < 0.001) and exhibited higher comorbidity, increased prevalence of pre-fracture dementia (31.8% vs. 21.1%, p = 0.001), and more severe frailty (0.14 vs. 0.11, p = 0.015). They also had greater mobility impairment and a higher proportion of extracapsular fractures (68.2% vs. 58.8%, p = 0.014). Discharge disposition differed significantly: “Never users” were more frequently discharged home (52.8% vs. 34.8%), whereas “Ever users” more commonly transitioned to rehabilitation facilities (56.9% vs. 38.1%).

**FIGURE 1:**
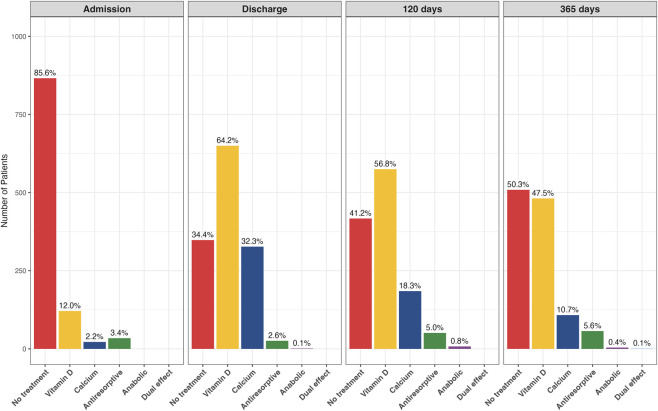
Prevalence of osteoporosis treatments across all time points. Bar plot illustrating the distribution of osteoporosis treatments at admission, discharge, 120 days, and 365 days. Each bar represents the proportion of patients receiving no treatment or a treatment from a specific osteoporosis medication. As patients may receive multiple treatments concurrently, cumulative percentages exceed 100% at each time point.

Figure 1 shows the prevalence of each type of osteoporosis treatment over time, alongside the proportion of untreated patients. Upon admission, most of them (85.6%) were untreated. At discharge, the proportion of untreated patients declined, reaching a minimum of 34.4%, rising again to approximately 50% at 365 days post-surgery. Vitamin D and calcium were the most used treatments, with usage peaking at discharge (64.2% and 32.3%, respectively). In contrast, antiresorptive, anabolic, and dual effect agents were rarely used. Antiresorptive use declined from 3.4% upon admission to 2.6% at discharge, then rose up to 5.6% at 365 days post-surgery. Use of anabolic and dual effect agents remained below 1% throughout the overall study period.

**FIGURE 2:**
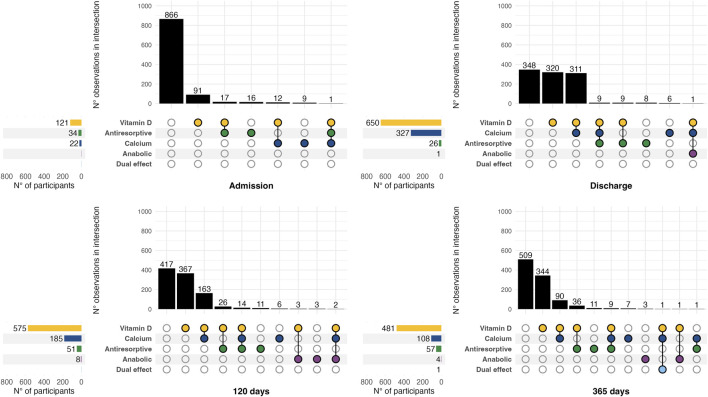
Upset plot showing monotherapy and combination regimens of osteoporosis treatments across all time points. Upset plot of osteoporosis treatments at admission, discharge, 120 days, and 365 days. Single dots indicate individual treatments; connected dots represent combination regimens. Vertical bars show the number of patients per regimen, and horizontal bars indicate the absolute frequency per treatment.

Figure 2 provides a detailed overview of osteoporosis treatment, including monotherapy and combination regimens, upon admission, at discharge, and at follow-up. Across all time points, most patients were treated with vitamin D and/or calcium alone. These regimens accounted for 76.7% (n = 112/146) upon admission, 95.9% (n = 637/664) at discharge, 90.1% (n = 536/595) at 120 days post-surgery, and 87.7% (n = 441/503) at 365 days post-surgery. In contrast, antiresorptive, anabolic, and dual effect agents were infrequently used and typically administered alongside supplements rather than alone. As shown in Table 2, multivariable logistic regression identified male sex (OR 2.37, 95% CI 1.59–3.55; p < 0.001) as the only independent predictor of never receiving osteoporosis treatment.

**TABLE 2:** Clinical and socio-demographic factors associated with non-use of osteoporosis treatment (i.e., Never users).

Variables	OR (95% CI)	p-value
Gender		
*Female*	Reference	-
*Male*	2.374 (1.586; 3.554)	<0.001
Age (years)	1.023 (0.996; 1.050)	0.093
Frailty index*	1.043 (0.902; 1.205)	0.572
Type of fracture		
*Intracapsular-subcapital*	Reference	-
*Extracapsular*	0.978 (0.658; 1.456)	0.915
Destination at discharge		
*Home*	Reference	-
*Rehabilitation*	0.814 (0.532; 1.245)	0.343
*Nursing home*	1.092 (0.510; 2.337)	0.821
*Other wards*	0.479 (0.106; 2.169)	0.340

* FI, was multiplied by 10.

## 4 Discussion

In this observational prospective study, involving 12 Orthogeriatric Italian wards, osteoporosis treatment was found to be markedly suboptimal both before and after HF surgery. Upon admission, only 1.5 out of ten patients were receiving osteoporosis treatment, most commonly limited to vitamin D and calcium supplementation. Hospitalization provided an opportunity to initiate treatment, with prescriptions effectively rising at discharge. However, the proportion of patients undergoing treatment declined substantially over time, with roughly half of patients on treatment at 1 year. Antiresorptive, anabolic, or dual effect agents were rarely taken during the study period, with only a modest increase observed in the year after surgery, highlighting the limited use of active pharmacological therapies beyond basic supplementation.

Clinical guidelines recommend pharmacological treatment for the secondary prevention of HF, including antiresorptive agents, anabolic therapies, or dual effect drugs, alongside vitamin D and calcium to maximize the efficacy of osteoporosis-specific therapies (Gregson et al., 2022; Yang et al., 2024; Nakamura et al., 2017; Tarantino et al., 2017). Our findings reveal a clear gap between these recommendations and real-world practice in Italy. Despite their high-risk profile, HF patients in our cohort were substantially undertreated both upon admission and during follow-up. Comparable results have been reported elsewhere, with post-fracture treatment rates ranging from 16.9% in a Turkish cohort of 552 patients over 50 years to 23.2% in the study by Gonnelli et al. following HF surgery (Gonnelli et al., 2017; Çakır et al., 2025). By contrast, data from the UK’s National Hip Fracture Database showed a marked improvement in osteoporosis treatment rates between 2016 and 2020 (Mohsin et al., 2023). Among patients not receiving any oral treatment at the time of HF admission, approximately one-third were prescribed osteoporosis medication at discharge, while an additional 13% received an injectable agent, resulting in nearly one in two patients being treated at discharge (Mohsin et al., 2023). These data suggest that where structured quality improvement programs—such as audits and national registries—are in place, substantial progress can be achieved in narrowing the treatment gap and improving clinical practice (NHFD, 2025).

Previous studies have also documented significant sex disparities in osteoporosis management after HF (Kirk et al., 2024; Kiebzak et al., 2002; Liu et al., 2016). Kiebzak et al. showed that men were significantly less likely than women to receive treatment, both at hospital discharge (4.5% vs. 27%) and at long-term follow-up (27% vs. 71%), with prescriptions often limited to calcium and vitamin D supplementation (Kiebzak et al., 2002). Similarly, in our study, male sex was associated with lower odds of initiating osteoporosis treatment. These findings highlight that osteoporosis in men is undertreated, even after a major sentinel event such as HF (Osteoporosis Foundation, 2025). This gap is partly sustained by the widespread yet misleading perception of osteoporosis as a “women’s disease”, despite evidence that fragility fractures in men carry substantially higher mortality (Fuggle et al., 2024; Katsoulis et al., 2017).

According to previous studies, advanced age and frailty are additional factors contributing to under-prescription of osteoporosis treatments (Giangregorio et al., 2006; Solomon et al., 2003; Chattaris et al., 2022). In a systematic review of 35 studies, four reported that older patients were more likely to be diagnosed with osteoporosis but less likely to receive treatment compared to younger individuals (Giangregorio et al., 2006). However, our findings do not confirm this association. The advanced age of our cohort and the limited variability in age distribution may have reduced our ability to detect age-related differences due to a ceiling effect. As for frailty, a retrospective study of 29,904 patients found that 51.3% were classified as frail according to a 93-item FI, yet only 7.2% received osteoporosis treatment during hospitalization (Chattaris et al., 2022). Notably, treatment lasting more than 90 days was associated with a similar reduction in subsequent fracture risk in both frail and non-frail patients, suggesting the benefit of these drugs for all patients, regardless of frailty status (Chattaris et al., 2022). In our cohort, frailty was not significantly associated with under-prescription of osteoporosis treatments. The discrepancy from previous findings may reflect the fact that most prescribers in our study were geriatricians, who are less likely to view frailty as a barrier to treatment (Shafiee et al., 2024). Additionally, our FI, which included fewer items than those used in the above-mentioned study, may have had lower sensitivity in detecting such an association.

Findings from our study have clinical implications. Many older adults who sustain HF likely have underlying osteoporosis and should have initiated treatment prior to the fracture (McCloskey et al., 2021; Keaveny et al., 2024). This highlights the need for a nationwide public health campaign on the under-prescription of osteoporosis therapies. The persistence of suboptimal treatment rates across the study period underscores the importance of integrated, multidisciplinary approaches to secondary prevention of HF. Such strategies should involve endocrinologists, orthopedic surgeons, and geriatricians during inpatient care, and engage general practitioners in the community to ensure continuity of care after discharge. Structured models such as Fracture Liaison Services (FLS) could play a pivotal role in bridging this therapeutic gap both during hospitalization and post-discharge, improving adherence and optimizing long-term outcomes (Li et al., 2021; Ruggiero et al., 2022).

The strengths of our study include its multicenter, national, and longitudinal design, as well as a large, well-characterized cohort with follow-up assessments at four time points over 1 year.

We also acknowledge several limitations. First, osteoporosis treatment was assessed only at discrete time points, without capturing events that might have occurred between assessments, such as medication adherence, reasons for discontinuation or switching, or prevalent or incident contraindications. Therefore, our findings should be interpreted as reflecting treatment prevalence at predefined time points rather than continuous treatment exposure.

In addition, data on the underlying etiology of osteoporosis, associated clinical risk factors (e.g., lifestyle habits, concurrent medications, nutritional status, genetics), bone mineral density, and reasons for not receiving osteoporosis treatment (e.g., contraindications or patient refusal) were not available, limiting our ability to assess factors potentially contributing to the observed treatment gap. Moreover, the lack of detailed hip fracture history (including fracture recurrence and trauma energy) limited our ability to assess patient risk. Furthermore, analyses were restricted to patients with available osteoporosis therapy data at all four time points; consequently, individuals who died or were lost to follow-up were not included, which may have introduced survivorship and attrition bias. Residual confounding by unmeasured factors cannot be excluded. Additional subgroup analyses were not performed due to sample size constraints and the exploratory nature of the study; this should be considered in future research. Finally, although the number of participating centers was substantial, they did not cover the entire Italian territory, potentially limiting the generalizability of the results.

## 5 Conclusion

Osteoporosis remains a leading cause of fragility fractures, significantly affecting morbidity, mortality, and healthcare costs. Early and effective treatment is pivotal to preventing the “fracture cascade” and its consequences, particularly in older adults. Our multicenter study confirms persistent treatment gaps, similar to findings in countries without a structured national program for HF registry. Future efforts should prioritize the implementation of structured care models for HF patients and the establishment of a national registry to systematically monitor prescription patterns and adherence. Such initiatives are essential to improve patient outcomes and reduce the overall burden of osteoporotic fractures on healthcare systems.

## Collaborators of the Gruppo Italiano di Ortogeriatria (GIOG) study group

Alice Rivolta, Luca Tinelli, Elena Page, Martina Marelli, Leonardo Barbieri, Giorgio Mauri, Alberto Saporito, Camilla Tocci (School of Medicine and Surgery University of Milano-Bicocca); Emanuela Rossi (Bicocca Center of Bioinformatics, Biostatistics and Bioimaging University of Milano-Bicocca); Maurizio Corsi (Acute Geriatric Unit IRCCS San Gerardo dei Tintori Foundation); Giuseppe Castoldi, Francesca Colombo, Luca Molteni (Orthopedics and Traumatology Unit ASST della Brianza P.O. Carate Brianza); Maria Lia Lunardelli, Chiara Bendini (Policlinico S. Orsola-Malpighi Bologna); Caterina Trevisan, Pierfederico Scaroni (Department of Medical Science University of Ferrara); Alice Ceccofiglio, Alessandro Cartei, Gaia Rubbieri, Giulio Mannarino (Careggi University Hospital of Florence); Enrico Benvenuti, Simone Pupo, Silvia Tognelli, Chiara Bandinelli (Azienda USL Toscana centro); Emilio Martini, Elisa Crocetti, Elena Sperti (Orthogeriatric Unit University of Modena and Reggio Emilia); Monica Pizzonia (Orthogeriatric Unit, IRCCS Ospedale Policlinico San Martino, Genoa); Luca Tagliafico, Stefania Peruzzo (University of Genoa); Alberto Pilotto, Antonella Barone, Alberto Cella (Galliera Hospital Genova); Chiara Ceolin, Labjona Haxhiaj, Cristina Simonato (Department of Medicine University of Padua); Antonio De Vincentis, Andrea Cavalli, Alice Laudisio (Campus Bio-Medico University Rome); Filippo Fimognari (Azienda Ospedaliera di Cosenza SS. Annunziata); Paola Cena, Martina Bonetto, Maria Garro (Santa Croce e Carle Hospital Cuneo); Andrea Rossi (Ospedale Ca’ Foncello AULSS 2, Treviso).

## Data Availability

The datasets presented in this article are not readily available because individual participant data cannot be made publicly available due to the sensitive nature of the personal health data and privacy and confidentiality restrictions. However, data may be made available for statistical and scientific research purposes upon reasonable request and subject to appropriate approvals. Requests to access the datasets should be directed to w.guo@campus.unimib.it.
