# Supplementary material for: Underuse of osteoporosis treatments before and after hip fracture: longitudinal findings from the Gruppo Italiano di Ortogeriatria (GIOG 2.0) study
Source: Front Aging. 2026 May 29;7:1837212. doi: 10.3389/fragi.2026.1837212 (PMC13260223; doi:10.3389/fragi.2026.1837212)
Supplement: Supplementary file 1 [file DataSheet1.pdf]

SUPPLEMENTARY MATERIAL

Supplementary Table 1. Variables included in the calculation of the Frailty Index

Diabetes mellitus	No = 0, without organ damage = 0.5, with organ damage = 1	Liver disease	No = 0, mild = 0.5, moderate-to-severe = 1
Leukemia, lymphoma or solid non metastatic tumor	No = 0, yes = 1	Solid metastatic tumor	No = 0, yes = 1
Acquired Immune-Deficiency Syndrome	No = 0, yes = 1	Moderate-to-severe renal failure	No = 0, yes = 1
Heart failure	No = 0, yes = 1	Coronary heart disease	No = 0, yes = 1
Chronic pulmonary disease	No = 0, yes = 1	Peripheral vascular disorder	No = 0, yes = 1
Cerebrovascular disorder	No = 0, yes = 1	Dementia	No = 0, yes = 1
Connective tissue disease	No = 0, yes = 1	Peptic ulcer	No = 0, yes = 1
Hemiplegia	No = 0, yes = 1	Psycholeptic drugs assumption at admission	No = 0, yes = 1
Dependence in hygiene	No = 0, yes = 1	Dependence in dressing	No = 0, yes = 1
Dependence in toileting	No = 0, yes = 1	Dependence in locomotion	No = 0, yes = 1
Incontinence	No = 0, yes = 1	Dependence in eating	No = 0, yes = 1
Mobility (SAHFE)	Able to walk independently = 0,<br>Able to walk outdoor with 1 aid = 0.25,<br>Able to walk outdoor with 2 aids = 0.5,<br>Able to walk outdoor only with help = 0.75,<br>Not able to walk = 1	Domiciliation at admission	Home = 0, Home with a family member/caregiver = 0.5, Nursing home = 1
